# Dietary Impact of Produce Prescriptions for Patients With Hypertension

**DOI:** 10.5888/pcd15.180301

**Published:** 2018-11-15

**Authors:** Erika S. Trapl, Samantha Smith, Kakul Joshi, Amanda Osborne, Michele Benko, Anna Thornton Matos, Shari Bolen

**Affiliations:** 1 Department of Population and Quantitative Health Sciences, Case Western Reserve University, Cleveland, Ohio; 2 Cuyahoga County Board of Health, Parma, Ohio; 3 Ohio State University, Cleveland, Ohio; 4 Department of Medicine, MetroHealth Medical Center/Case Western Reserve University, Cleveland, Ohio; 5 Center for Health Care Research and Policy, Case Western Reserve University at MetroHealth Medical Center, Cleveland, Ohio

## Abstract

**Introduction:**

Little is known regarding the impact of produce prescriptions within the context of hypertension visits at safety net clinics. We evaluated intervention effectiveness on patient usage of farmers markets and dietary change related to fruit and vegetable consumption.

**Methods:**

Health Improvement Partnership — Cuyahoga worked with 3 clinics to integrate, implement, and evaluated a produce prescription for hypertension (PRxHTN) program. PRxHTN involves 3 monthly, nonphysician provider visits, comprising blood pressure measurement, nutrition counseling, and four $10 farmers market produce vouchers, for hypertensive adult patients screening positive for food insecurity. Dietary measures were collected at visits 1 and 3. Voucher use was tracked via farmers market redemption logs.

**Results:**

Of the 224 participants from 3 clinics, most were middle-aged (mean age, 62 y), female (72%), and African American (97%) and had a high school education or less (62%). Eighty-six percent visited a farmers market to use their produce vouchers, with one-third reporting it was their first farmers market visit ever. Median number of farmers market visits was 2 (range: 0–6), and median number of vouchers redeemed was 8 (range: 0–12). Among the subsample with follow-up survey data (n = 137), significant improvement in fruit and vegetable consumption was observed as well as a decline in fast food consumption.

**Conclusion:**

PRxHTN participants visited at least 1 farmers market, reported increases in provider communication related to diet, and exhibited significant changes in dietary behavior. PRxHTN can serve as a strong model for linking safety net clinics with farmers markets to promote community resource use and improve fruit and vegetable consumption among food-insecure patients with hypertension.

## Introduction

Eating a diet rich in fruit and vegetables lowers risk of mortality from cardiovascular diseases (1). In 2015, 12% of adults in the United States met recommendations for eating fruit and 9% of adults met recommendations for eating vegetables (2). These trends are much worse among those of lower socioeconomic status (2).

Farmers markets are a strategy to improve fruit and vegetable consumption (3), and exposure to farmers markets increases fruit and vegetable consumption among low-income populations (4). However, purchasing more costly produce instead of inexpensive processed foods remains a challenge for those at economic disadvantage. By one estimate, low-income households would have to allocate 43% to 70% of their budget to meet dietary guidelines for fruit and vegetable intake compared with 15% to 18% of the budget of average households (5). Despite reliance on the Supplemental Nutrition Assistance Program (SNAP) and other food assistance programs, access to fresh, nutrient-rich foods remains a challenge for low-income households (6,7).

Produce prescription partnerships that engage public health, health care clinics, and farmers markets are one approach gaining momentum to improve fruit and vegetable intake (8–13). A “prescription” refers patients to community resources providing fruit and vegetable access. These community-linked, health care provider–assisted models serve as powerful tools for motivating behavior change (14) and increasing fruit and vegetable consumption among low-income persons (10,12).

Among underserved populations with chronic conditions exacerbated by poor diet, little evidence exists on the impact of these interventions. Although decreases in glycated hemoglobin A_1c_ were observed among people with diabetes, participants’ weight and blood pressure remained unchanged, and redemption and dietary behaviors were not examined (12). Others have demonstrated reduction in body mass index among low-income urban patients with chronic disease in a case–control design; however, it is unclear what effect the program had on intermediate outcomes such as fruit and vegetable consumption (13). We sought to evaluate the effect of a brief clinical produce prescription intervention for food-insecure patients with hypertension on program participation, nutrition counseling, fruit and vegetable voucher redemption, and dietary behavior change.

## Methods

### Study design and implementation

We conducted a comprehensive evaluation of a produce prescription program for patients with hypertension (PRxHTN). PRxHTN represents a clinical–community linkage intervention aiming to increase fruit and vegetable consumption among safety net clinic patients with hypertension who are at risk for food insecurity by providing incentives to use local farmers markets via produce prescription vouchers.

PRxHTN was implemented by partners of a countywide health collaborative, Health Improvement Partnership — Cuyahoga (HIP-Cuyahoga; hipcuyahoga.org), in response to a Centers for Disease Control and Prevention (CDC) grant that used mechanisms to manage hypertension at both an individual and a clinic population level. Details on the partnerships, planning process, and overall framework for program implementation at clinical sites are reported elsewhere (11). The MetroHealth Medical System Institutional Review Board approved the study.

### Sites, population, and intervention

Three safety net clinics from 3 separate health systems were recruited to offer PRxHTN in spring 2015. Sites were selected based on their location and their focus on delivering primary care to underserved populations. Seven nonphysician health care providers from the clinics (2 to 3 per site) were trained in program delivery. Twenty farmers markets agreed to participate in PRxHTN by accepting vouchers and logging redemptions. Details on trainings for providers and farmers market managers are provided elsewhere (11).

PRxHTN was modeled after a program serving low-income pregnant women with young children (PRxMoms) (10). PRxMoms engaged prenatal programs in providing nutrition education, resources, and up to 4 months of farmers market produce vouchers to low-income pregnant women. The program was informed by the theory of implementation intentions and repeated behaviors, which stresses the importance of developing plans that address the when, where, and how of achieving a decided goal (15). In response to our CDC funding opportunity, key components of PRxMoms were translated into a chronic disease care model and adapted for an underserved adult population with diagnosed hypertension.

By using a brief screening tool, providers identified patients based on age (adults 18 or older), hypertension diagnosis, and screening positive on a validated 2-item screener for food insecurity (16), yielding a convenience sample drawn from patients scheduled for appointments during the recruitment period. PRxHTN was offered to align with an evidence-based best practice for hypertension management implemented at the clinics (17). Each PRxHTN visit (3 total; 1 per month) involved a blood pressure measurement, targeted nutrition counselling, and providing four $10 vouchers to purchase fresh produce only at farmers markets. To support participants’ shopping habits, they had the flexibility of redeeming vouchers all at once or over time at any participating farmers market. Participants set goals around increasing fruit and vegetable consumption and identified motivations for changing behavior at each visit. Providers reviewed the following educational materials with the participants: 1) location card for 20 farmers markets accepting PRxHTN vouchers; 2) Community Food Guide, providing guidance on low-cost healthy meal plans, fresh food storage tips, and seasonal Ohio fruit and vegetables (18); and 3) adapted handouts on Dietary Approaches to Stop Hypertension, the DASH diet (19). Recruitment occurred from June through September 2015 and the program was conducted between July and December 2015 to align with the farmers market season; goal enrollment was 75 patients per site based on available resources for farmers market vouchers.

### Data collection

Data were collected from 2 sources: patients and farmers markets. Patients completed an intake survey during visit 1 and a postprogram survey at visit 3. During each visit, participants received a produce prescription, which documented each patient’s reasons for the prescription and their dietary behavior goals. All data collection instruments were coded with a unique identification number, which was recorded in the electronic health record. This identification number was used on the PRxHTN vouchers so that data could be linked for analysis. Vouchers received by farmers markets were considered redeemed, and the number of redeemed vouchers was recorded for each participant.

### Measures

At intake, participants were asked to report sex, age in years, racial/ethnic identity, highest level of education, number of adults and children in the home, number of years since hypertension diagnosis, and whether they were currently receiving SNAP benefits.

At postprogram (visit 3), participants assessed impact of the program, including increased visits to farmers markets, trying a new farmers market, greater importance of fruit and vegetable consumption, intention to shop at farmers markets in the future, and trying new fruit and vegetables.

When completing the prescription voucher with the provider, participants’ goals and reasons to use the prescription included the following: increase fruit and vegetable servings; shop more frequently for fruit and vegetables; visit farmers markets more frequently; add fruit and vegetables to meals and snacks; try new fruit and vegetables; improve hypertension; lead a healthier lifestyle; have a healthy family; find a new place (farmers market) to buy fruit and vegetables; and reduce risk of chronic disease. Participants could choose more than 1 reason or goal; responses were coded as selected (1) or not selected (0).

At intake, participants were asked about perceived barriers to eating fruit and vegetables, general perceptions of farmers markets, and their current food shopping habits. Barriers were coded as yes/present or no. Farmers market perceptions were coded on a 5-point Likert scale (strongly agree to strongly disagree) and included items such as “Quality of fruit and vegetables at farmers markets is as good or better than the grocery store.” Responses were recoded to reflect agreement (agree or strongly agree) for each item. For shopping behaviors, participants were asked (yes/no) if they had ever shopped at a farmers market, the types of food stores they had shopped at in the last month, and if they use an electronic benefits transfer card or food stamps. Household responsibility for food shopping and meal preparing was also assessed by using a 5-point Likert scale (none to all).

Two items assessed patient–provider communication around diet on both the intake and postprogram survey; responses ranged from never to always.

Fruit and vegetable voucher redemption data were collected from each farmers market showing farmers market name, date of the redemption, and dollar amount redeemed.

Participants’ fruit and vegetable consumption was assessed using the Fruit and Vegetable Checklist (20). This validated tool includes 7 items, facilitating computation of daily servings of fruit and daily servings of vegetables individually. We assessed fast food consumption by asking how many days of the past week the participant had eaten fast food, with responses ranging from 0 to 7.

### Analyses

Participant demographic characteristics, goals, perceptions, and food-related shopping behaviors were examined by using descriptive statistics. Bivariate analyses compared completers (ie, those with a visit 3/postprogram survey) and noncompleters (ie, those without) by using χ^2^ tests. Change in self-reported nutrition counseling frequency was assessed by using nonparametric tests. PRxHTN voucher use at farmers markets was calculated at the participant level and aggregate level. Changes in fruit and vegetable and fast food consumption were evaluated using paired *t* tests. Significance was set at *P* < .05 for all analyses; final analyses using SPSS v.24 (IBM, Inc) were conducted in 2018.

## Results

Overall, 266 patients were screened and 224 enrolled in PRxHTN from 3 clinics (Table 1). Most were African American/black (97%) and women (72%) and had a high school or general equivalency diploma or less (62%). Mean (standard deviation [SD]) age was 62 (11) years and years with hypertension was 13 (12). Approximately half were receiving SNAP benefits (48%). Mean (SD) daily fruit servings was 1.7 (1.4) and mean (SD) daily vegetable servings was 1.7 (1.3); fast food was consumed a mean (SD) of 1.5 (1.5) days per week. Program follow-up rates were 81% (n = 182) at check-in (visit 2) and 61% (n = 137) at postprogram (visit 3). Generally, participants with a postprogram survey were similar to those without a postprogram survey.

**Table 1:** Baseline Characteristics of Patients in the Produce Prescription for Hypertension Program, Cuyahoga County, Ohio, 2015

Characteristic	Enrolled, n = 224	Completed, n = 137
**Demographic characteristics**		
**Age, mean (standard deviation)^a^, y**	61.6 (11.2)	60.3 (10.9)
**Female, %**	71.9	71.1
**African American/black, %**	96.8	98.5
**Education, %**		
Less than high school or general equivalency diploma	22.1	19.2
High school or general equivalency diploma	39.4	41.5
Some college	23.5	24.6
College degree	15.0	14.6
**No. of adults in home, mean (standard deviation)**	1.7 (0.8)	1.6 (0.8)
**No. of children in home, mean (standard deviation)**	0.7 (1.1)	0.6 (1.0)
**Years with hypertension, mean (standard deviation)**	13.1 (11.6)	13.2 (10.9)
**Receives Supplemental Nutrition Assistance Program benefits, %**	48.1	49.6
**Dietary behaviors**		
Daily fruit consumption, mean (standard deviation)	1.7 (1.4)	1.6 (1.3)
Daily vegetable consumption, mean (standard deviation)	1.7 (1.3)	1.7 (1.1)
Fast food consumption (days per week), mean (standard deviation)	1.5 (1.5)	1.4 (1.4)

a Significant difference between participants with and without a postprogram survey (*P* = .04).

Of those completing PRxHTN (n = 137), 88% indicated they visited farmers markets more than before PRxHTN, 82% tried a new farmers market, and 95% reported that they would continue to shop at farmers markets in the future. Additionally, 88% reported that eating fruit and vegetables was more important because of the program, and 82% had tried a new fruit or vegetable.

### Goals, barriers, perceptions, and food shopping behaviors

Program completers and noncompleters overwhelmingly endorsed goals of increasing fruit and vegetable consumption and improving hypertension (Table 2). Significant differences were observed for shopping more frequently for fruit and vegetables, adding fruit and vegetables to meals and snacks, and finding a new place to buy fruit and vegetables such that completers endorsed these goals more than noncompleters. In both groups, financial barriers to fruit and vegetable consumption were most highly endorsed; however, this concern was significantly higher among completers. There were no significant differences in perceptions of farmers markets except completers reported interest in shopping at farmers markets at a higher rate than noncompleters. Completers demonstrated different food shopping behaviors compared with noncompleters; they were significantly less likely to have shopped at a supermarket, grocery store, supercenter, or warehouse in the past month, and their use of convenience stores (*P* = .07) and food pantries or shelters (*P* = .05) were marginally but not significantly higher.

**Table 2:** Goals, Barriers, Farmers Market Perceptions, and Food-Related Shopping Behaviors in the Produce Prescription for Hypertension Program, Cuyahoga County, Ohio, 2015

Category	Completed Program, % (n = 137)	Did Not Complete, % (n = 87)	*P* Value^a^
**Goals and reasons for participating in the produce prescription for hypertension program^b^**			
Increase fruit and vegetable servings	97.1	98.9	.65
Shop more frequently for fruit and vegetables	32.8	20.7	.049
Visit farmers market more frequently	86.9	79.3	.13
Add fruit and vegetables to meals and snacks	53.3	37.9	.03
Try new fruit and vegetables	35.0	41.4	.34
Improve hypertension	95.6	95.4	>.99
Lead a healthier lifestyle	81.8	79.3	.65
Have a healthy family	40.1	40.2	.99
Find new place to buy fruit and vegetables	50.4	35.6	.03
Reduce risk of chronic disease	78.8	71.3	.20
**Barriers to fruit and vegetable consumption^b^**			
Lack of access to fruit and vegetables in neighborhood	39.4	31.0	.20
Limited or no storage space for fruit and vegetables	14.6	12.6	.68
Don’t like fruit and vegetables	5.8	4.6	.77
Family doesn’t like fruit and vegetables	1.5	1.1	>.99
Not enough time	5.8	9.2	.34
Fruit and vegetables are expensive	69.3	51.7	.008
**Farmers market perceptions^c^**			
Interested in shopping at a farmers market	100.0	91.4	.001
Have transportation to get to a farmers market	80.3	84.8	.42
Quality of fruit and vegetables at farmers markets is as good or better than a grocery store	80.3	80.9	.93
Wide variety of fresh produce is available at farmers markets	86.8	88.1	.81
Prices at farmers markets are affordable	69.4	66.2	.65
**Food shopping behaviors**			
**Ever shopped at a farmers market**	66.2	56.1	.14
**Food stores where shopped in past month**			
Supermarket, grocery store, supercenter, or warehouse	90.5	97.7	.04
Convenience or dollar variety store	26.3	16.1	.07
Famers market	10.3	7.0	.40
Food pantry or shelter	29.9	18.4	.05
**Use Supplemental Nutrition Assistance Program electronic benefits transfer card**	49.6	45.7	.58
**Responsible for majority of food shopping for household^d^**	70.1	60.5	.14
**Responsible for majority of meal preparing for household^d^**	70.1	60.9	.16

a χ^2^ test.

b Participants could choose more than 1 goal and reason or barrier.

c Percentage that agreed or strongly agreed, coded by using a 5-point Likert scale (strongly agree to strongly disagree).

d Percentage responding “more than half” or “all” on a 5-point Likert scale (none to all).

### Voucher redemption and farmers market visits

Participant-level voucher redemption data were available for patients enrolled at only 2 of the 3 clinics (n = 149) because of a reporting error on the part of the third clinic. Of those, 86% of participants visited at least 1 participating farmers market and redeemed at least 1 voucher; one-third reported visiting a farmers market for the first time ever during the program. Median number of farmers market visits was 2, with a range of 0 to 6. Median number of vouchers redeemed was 8 (representing $80 worth of fruit and vegetables), and the maximum redeemed was 12 (or $120, the maximum amount provided to participants). Total fruit and vegetable sales at participating farmers markets from PRxHTN vouchers, obtained for the full patient sample (n = 224), was $15,140. Overall, 12 of 20 farmers markets were patronized.

### Dietary counseling and behavior change

Among the 137 participants with intake and postprogram survey data, self-reported frequency of nutrition counseling during health care visits significantly increased from baseline to visit 3 (*P* < .001). Patients reporting that their health care team “always” talked about their overall diet increased from 41% to 65%, while reporting that their health care team “always” talked about increasing their daily fruit and vegetable consumption and variety increased from 38% to 75% (Table 3).

**Table 3:** Intake and Postprogram Communication and Dietary Behavior Among Program Completers in the Produce Prescription for Hypertension Program, Cuyahoga County, Ohio, 2015

Behavior	No.	Intake	Postprogram	*P* Value
Health care team “always” talks about overall diet, %^a^	122	41.0	64.8	<.001^b^
Health care team “always” talks about increasing fruit and vegetable consumption, %^a^	121	38.0	75.2	<.001^b^
Daily servings of fruit, mean (standard deviation)^c^	125	1.6 (1.3)	2.4 (1.2)	<.001^d^
Daily servings of vegetables, mean (standard deviation)^c^	126	1.7 (1.1)	2.5 (1.3)	<.001^d^
No. days ate fast food in past week, mean (standard deviation)	129	1.3 (1.4)	0.7 (1.0)	<.001^d^

a Responses on a 5-point Likert scale from never to always.

b Assessed by using nonparametric tests.

c Assessed by using the Fruit and Vegetable Checklist (20).

d Paired *t* test.

Significant changes in dietary behavior were also observed among participants with follow-up (Table 3). Daily fruit consumption increased from a mean (SD) of 1.6 (1.3) servings to 2.4 (1.2) servings (*P* < .001), and daily vegetable consumption increased from a mean (SD) of 1.7 (1.1) servings to 2.5 (1.3) servings (*P* < .001). Farmers market visits and voucher redemption were not associated with fruit and vegetable consumption. Fast food consumption significantly decreased from a mean of 1.3 days per week to 0.7 days per week (*P* < .001).

## Discussion

PRxHTN engaged food-insecure, urban residents with hypertension in using an existing community resource of farmers markets to make recommended lifestyle changes. This was executed through a brief intervention during clinical visits with an existing nonphysician health care team member, allowing for an appropriate venue within which to discuss health-related benefits of dietary change and the practicalities of addressing barriers to dietary changes through providing relevant information and vouchers to purchase fresh, local fruit and vegetables at farmers markets.

Our findings extend the current literature by documenting significant intermediate dietary outcomes among patients with hypertension experiencing food insecurity. Overall, those who visited at least 1 farmers market reported a significant increase in provider communication related to diet and fruit and vegetable consumption and a decline in fast food consumption. Participants completing the program reported consuming a combined average of 4.9 servings of fruit and vegetables per day, effectively reaching the daily recommendation of 5 servings of fruit and vegetables compared with 3.3 at baseline. Although fast food consumption was not a primary focus of the program, a focus on reducing sodium along with increasing intake of fruit and vegetables may have contributed to changes in this behavior.

Our work highlights that among this particular population, patients are willing to set goals to improve their health condition, including increasing fruit and vegetable consumption and shopping at farmers markets. Participants had been living with diagnosed hypertension for over a decade on average and had likely received counseling on the benefits of lifestyle changes to improve hypertension. Notably, only 5% of participants indicated that they did not like fruit and vegetables. Two main barriers reported by participants included financial constraints and lack of access to fruit and vegetables in their neighborhoods. PRxHTN sought to address both of these barriers by promoting use of neighborhood farmers markets and providing financial resources to relieve the budgetary strain of purchasing fresh fruit and vegetables. Although these results are promising, it is unclear if the observed changes were maintained without ongoing access to additional financial resources.

Previous work has indicated that providers particularly appreciate that produce prescription programs allow them to provide resources that enable their clients to act on the lifestyle change advice they offer (10,21). Having an opportunity to provide such resources to clients may have facilitated counseling conversations about lifestyle behavior change and may have contributed to the significant increases in provider communication related to diet and fruit and vegetable consumption reported by participants. Given the new American College of Cardiology and American Heart Association hypertension guideline (22), which places a larger emphasis on lifestyle modification for management of hypertension, programs like PRxHTN are critically necessary for both management and prevention of hypertension as they are well positioned to deliver content while promoting healthy behavior change.

This work may have broader implications for other chronic diseases that recommend dietary changes for prevention and management. Although the longer-term goal is improved chronic disease outcomes, our work documents intermediate steps of understanding whether a brief intervention can affect dietary behaviors among people experiencing food insecurity. PRxHTN changes dietary behavior among people living with hypertension. This program may produce the same increases in fruit and vegetable consumption among food-insecure people who are being treated for other chronic diseases.

There are notable limitations to our study. First, the overall sample size was modest, although 3 different clinics from 3 separate health systems were represented, and only 61% of enrolled participants attended the third visit. While those who completed the program were similar to the enrolled population demographically, completers were more likely to report cost of fruit and vegetables as a significant barrier to fruit and vegetable consumption as well as interest in shopping at farmers markets. Thus, those who continue to participate may be in greatest need of additional financial resources and food access to support dietary change. Further, without a control group, it is unclear whether changes in dietary behavior would have occurred with provider advice alone. However, providers often do not have time to counsel patients on fruit and vegetable consumption, and traditional nutrition counseling referrals and uptake remained low at these clinics during the study. Second, this program aligned with the local farmers market season such that vouchers were distributed when farmers markets tended to have a broader range of fruit and vegetables in season and available. Although some farmers markets are moving toward a year-round schedule, many farmers markets in this community do not have a sufficient supply of fresh produce to make a year-round farmers market feasible. Thus, a shortcoming of PRxHTN is its reliance on seasonal farmers markets to address physical access to fresh fruit and vegetables. Given the high proportion of participants who reported shopping at a grocery store in the past month, it may be worthwhile to extend the program to these store types that are open year-round to allow participants to maintain their fruit and vegetable purchasing and consumption practices. Finally, programs such as PRxHTN require significant funding to support the cost of fruit and vegetable vouchers, require staff time to coordinate program roll-out, and assume existence or development of a strong farmers market presence. To date, PRxHTN has relied on time-limited local foundation funding and limited federal funding. Long-term sustainability and expansion of this model requires innovative approaches to dedicated funding to offset the cost of program coordination staff and fruit and vegetable vouchers or alternative methods to securing free fresh produce.

People with hypertension who are simultaneously experiencing food insecurity may be unable to execute recommended dietary changes because of physical and financial access barriers. PRxHTN serves as a strong model for linking safety net clinics with local farmers markets to promote community resources and improve fruit and vegetable consumption among this population.
